# A comprehensive ethnic-based analysis of alpha thalassaemia allelle frequency in northern Thailand

**DOI:** 10.1038/s41598-017-04957-2

**Published:** 2017-07-05

**Authors:** Mattapong Kulaphisit, Jatupol Kampuansai, Kamonlak Leecharoenkiat, Methi Wathikthinnakon, Daoroong Kangwanpong, Thongperm Munkongdee, Saovaros Svasti, Suthat Fucharoen, Duncan R. Smith, Pathrapol Lithanatudom

**Affiliations:** 1 Center of Excellence in Bioresources for Agriculture, Industry and Medicine, Department of Biology, Faculty of Science, Chiang Mai University, Chiang Mai, 50300 Thailand; 2 Department of Clinical Microscopy, Faculty of Allied Health Sciences, Chulalongkorn University, Bangkok, 10330 Thailand; 3 Molecular Pathology Laboratory, Institute of Molecular Biosciences, Mahidol University, Nakornpathom, 73170 Thailand; 4 Thalassemia Research Center, Institute of Molecular Biosciences, Mahidol University, Nakornpathom, 73170 Thailand

## Abstract

Alpha (α)-thalassaemia is one of the most prevalent hereditary blood disorders, commonly affecting Southeast Asian people, with the highest incidence (30–40%) being seen in northern Thailand. However, this high incidence was estimated without consideration of the variations between ethnic populations and the geographical location of the populations. To address this issue, a total of 688 samples from 13 different northern Thai ethnic groups (30 villages) categorized into three linguistic groups were genotyped for deletional alpha-thalassaemia (-α^3.7^, -α^4.2^, --^SEA^ and --^THAI^) and/or non-deletional alpha-thalassaemia (α^CS^ and α^PS^) via multiplex gap-PCR and dot-blot hybridization, respectively. Alpha^+^(-α^3.7^, -α^4.2^, α^CS^ and α^PS^) and alpha°-thalassaemia (--^SEA^ and --^THAI^) allele frequencies (with 95% Confidence Interval) were the highest in the Sino-Tibetan group [0.13 (0.08–0.18)] and the Tai-Kadai group [0.03 (0.02–0.05)], respectively. With regards to ethnicity, the varying allele frequency of α^+^ and α°-thalassaemia amongst a variety of ethnic groups was observed. The highest α^+^-thalassaemia allele frequency was found in the Paluang [0.21 (0.10–0.37)] while α°-thalassaemia allele frequency was the highest in the Yuan [0.04 (0.01–0.10)]. These detailed results of alpha thalassaemia allele frequency and genetic diversity amongst the northern Thai ethnic groups demonstrate the need for ethnicity based thalassaemia prevention programs.

## Introduction

Alpha (α)-thalassaemia is a group of hereditary blood disorders that are found with very high prevalence in tropical and subtropical regions, and in particular in the people of South and Southeast Asian countries. The overall incidence of alpha-thalassaemia in Thailand appears to be unusually high, especially in the northern part where approximately 30–40% of residents have been reported to be either carriers or homozygotes^1^.

The inheritance of α-thalassaemia is autosomal recessive and a person with α-thalassaemia genotype could be either a carrier or a patient. Carriers are healthy, and as such α-thalassaemia is continuously maintained over generations. However, couples who are both carriers are likely to give birth to a child with α-thalassaemia associated with clinical symptoms^2, 3^. The α-globin genes located on chromosome 16p13.3 are responsible for α-globin production^4, 5^. Each haploid chromosome contains two copies of α-globin genes giving a total of four allelic copies in combination with the other homologous chromosome.

Alpha-thalassaemia is characterized by anomalies of the α-globin genes leading to reduced α-globin chain production, and α-globin is one of the major constituents of the haemoglobin of red blood cells. The reduction of α-globin chains in α-thalassaemia is most frequently caused by large deletions (-α^3.7^, -α^4.2^, --^SEA^ and --^THAI^), although non-deletional α-thalassaemia such as Hb Constant spring (α^CS^) and Hb Pakse (α^PS^) can occur^1, 6–9^. Presentation of α-thalassaemia is correlated with the number of α-globin genes affected. Loss of one (α^+^: -α^3.7^, -α^4.2^, α^CS^ and α^PS^) or two (α°: --^SEA^ and --^THAI^) α-globin gene/s on one chromosome generally presents as a silent carrier state, while loss of three (α^+^/α°) results in Hb H disease in which the pathology is primarily mediated by the relative excess of β-chains which can form tetramers of β-globin (β_4_) which can promote oxidative hemolysis. Loss of four α-globin genes (α°/α°) results in fatal Hb Barts’ hydrops fetalis syndrome. Where loss of three α-globin genes occurs through inheritance of a combination of deletional and non-deletional α-thalassaemia, presentation can be more severe than that which results from inheritance of deletional α-thalassaemia only, and consequently, the clinical symptom of an affected person with inherited α-thalassaemia alleles ranges from asymptomatic to blood transfusion-dependence to premature death of infants depending on the number of α-globin alleles affected^8^.

The morbidity and mortality of α-thalassaemia associated with significant clinical symptoms are therefore observed in haemoglobin H disease (Hb H, three missing functional α-globin alleles) and haemoglobin Bart’s hydrops fetalis syndrome (Hb Barts, a complete loss of functional α-globin alleles)^10^. In Thailand, due to the high prevalence of α-thalassaemia carriers, there is a significant number of patients with Hb H disease (7/1,000 newborns)^11^. More importantly, in northern Thailand, 0.33% of 52,625 fetuses were reported to be Hb Bart’s hydrops fetalis^12^. These confirm the necessity for accurate and effective management of α-thalassaemia in this part of the world.

Several α-thalassaemia surveys in the northern part of Thailand have demonstrated that there is a high (15–40%) prevalence of α-thalassaemia alleles in the northern Thai population^13, 14^. However, population sampling in most surveys was conducted on couples who went to hospitals for screening, so the prevalence observed was primarily determined from the overall population of the upper northern part of Thailand. Interestingly, a recent study that determined the prevalence of α-thalassaemia in a population-based study in the northern Thai population showed for the first time that the overall prevalence of α-thalassaemia in upper northern Thailand was 24% (33 of 141), and more importantly, the study highlighted the significantly different prevalence of α-thalassaemia amongst ethnic groups ranging from 0 to 50% of populations examined^15^. However, that study was limited by a low number of samples and sampling areas for some ethnic groups, and in particular, no hill-tribe groups belonging to the Sino-Tibetan and Hmong-Mien linguistic families were included in the study. To address these issues this study analysed a large cohort comprising of ethnic populations from numerous sampling areas throughout the northern part of Thailand including the northern minorities such as Shan, Karen and Htin. Thus, the objective of this study is to provide more comprehensive and meaningful data of common α-thalassaemia allele frequency in northern Thai people as well as in particular, in each ethnic population. This information will serve as a more practical basis for developing genetic counseling for the long-term effort to reduce the burden of Hb H and Hb Bart’s hydrops fetalis syndrome in the country.

## Results

**Figure 1:**
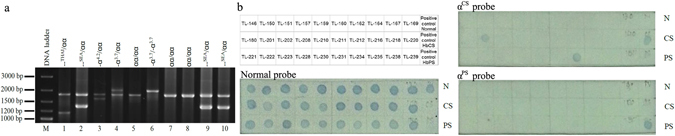
Six common α-thalassaemia types detected by multiplex-gap PCR and dot-blot hybridization techniques. (**a**) PCR products after alpha-globin gene analysis using the multiplex gap-PCR methodology, M = DNA marker, lane 1–4 = positive controls of alpha-globin heterozygotes which are --^THAI^/αα, --^SEA^/αα, -α^4.2^/αα and -α^3.7^/αα in order, lane 5 = negative control (αα/αα), lane 6 = unknown sample genotyped as -α^3.7^ homozygote, lanes 7–8 = unknown samples genotyped as normal and lanes 9–10 = unknown samples genotyped as --^SEA^ heterozygotes (A cropped gel is shown). The full-length gel is presented in Supplementary Figure S1. (**b**) Dot-blot hybridization analysis of the Lue ethnic group. Samples TL-201 and TL-234 were genotyped as α^CS^ heterozygotes. No samples were positive for α^PS^ (A cropped blot is shown). The full-length blot is presented in Supplementary Figure S2.

A total of 688 DNA samples from people belonging to 13 ethnic groups that are classified as part of three linguistic groups (Tai-Kadai, Austro-Asiatic and Sino-Tibetan) were analysed for four types of common deletional α-thalassaemias (-α^3.7^, -α^4.2^, --^SEA^, --^THAI^) by multiplex gap-PCR with nine specific primers for each type (Fig. 1a) and 350 of the total 688 samples were analysed for an additional two types of mutational α-thalassaemias (α^CS^ and α^PS^). Of the six common α-thalassaemia screened for, three different deletions (-α^3.7^, -α^4.2^, --^SEA^) and one point mutation (α^CS^) were found in this cohort.

The overall prevalence of the six common α-thalassaemia types assessed in this cohort of 13 ethnic groups is 19.51% (Table 1) with a frequency of 0.1008 (0.0788–0.1247) (Table 2). Almost all the α-thalassaemia detected in this study was heterozygous, except for one case of Hb H disease (-α^3.7^/--^SEA^) which was detected in one sample from the Yong ethnic group (Table 1).

**Table 1:** The number of affected person according to the genotype analysis with prevalence (%) of α-thalassaemia in the population residing in northern Thailand.

Ethnic group	Total sample	The analysis of alpha-thalassaemia genotype and prevalence (%)															Overall sample/prevalence (%) (1 + 2)
		No. sample*	-α^3.7^		-α^4.2^		--^SEA^	--^THAI^	Hb H	Total sample/prevalence	No. sample**	α^CS^		α^PS^		Total sample/prevalence (mutational type)^2^	
			-α^3.7^/αα	-α^3.7^/-α^3.7^	-α^4.2^/αα	-α^4.2^/-α^4.2^	--^SEA^/ αα	--^THAI^/ αα	(-α^3.7^/--^SEA^)	(deletional type)^1^		α^CS^ α/αα	α^CS^ α/ α^CS^ α	α^PS^α/αα	α^PS^ α/ α^PS^ α		
Yong	116	116	4/3.45	2/1.72	—	—	6/5.17	—	1/0.86	13/11.21	116	2/1.72	—	—	—	2/1.72	15/12.93
Lue	156	156	22/14.10	1/0.64	4/2.56	—	10/6.41	—	—	37/23.72	132	3/2.27	—	—	—	3/2.27	40/25.99
Yuan	48	48	6/12.50	—	—	—	4/8.33	—	—	10/20.83	18	2/11.11	—	—	—	2/11.11	12/31.94
Shan	53	53	11/20.75	—	—	—	4/7.55	—	—	15/28.30	ND	ND	ND	ND	ND	ND	15/28.30
Khuen	18	18	2/11.11	—	—	—	—	—	—	2/11.11	18	—	—	—	—	—	2/11.11
Htin	73	73	—	—	—	—	2/2.74	—	—	2/2.74	ND	ND	ND	ND	ND	ND	2/2.74
Paluang	19	19	8/42.11	—	—	—	—	—	—	8/42.11	19	—	—	—	—	—	8/42.11
Blang	20	20	1/5.00	—	—	—	1/5.00	—	—	2/10.00	20	—	—	—	—	—	2/10.00
Lawa	48	48	—	—	—	—	—	—	—	—	18	—	—	—	—	—	0
Mon	34	34	6/17.65	—	—	—	—	—	—	6/17.65	9	—	—	—	—	—	6/17.65
Skaw Karen	45	45	8/17.78	1/2.22	—	—	—	—	—	9/20.00	ND	ND	ND	ND	ND	ND	9/20.00
Pwo Karen	30	30	6/20.00	2/6.67	—	—	—	—	—	8/26.67	ND	ND	ND	ND	ND	ND	8/26.67
Padong Karen	28	28	4/14.29	1/3.57	—	—	—	—	—	5/17.86	ND	ND	ND	ND	ND	ND	5/17.86
**Total**	**688**	**688**	**78**/**11.34**	**7**/**1.02**	**4**/**0.58**	**0**	**27**/**3.92**	**0**	**1**/**0.15**	**117**/**17.51**	**350**	**7**/**2.00**	**0**	**0**	**0**	**7**/**2.00**	**124**/**19.51**

ND = Unverified point-mutational alpha-globin gene anomalies (α^CS^, α^PS^) by a dot-blot hybridization method.

*The number of sample enrolled in this study was subjected to four deletional alpha-thalassaemia screening (-α^3.7^, -α^4.2^, --^SEA^ and --^THAI^).

**The number of sample enrolled in this study was subjected to four deletional (-α^3.7^, -α^4.2^, --^SEA^ and --^THAI^) and two mutational (α^CS^, α^PS^) alpha-thalassaemia screening.

**Table 2:** The allele frequency of α-thalassaemia in the population residing in northern Thailand.

Linguistic group	Ethnic group	Total sample (*; **)	Alpha-thalassaemia allele frequency [Observed frequency (95% Confidence Interval, low-high)]									
			Deletional (α^+^)		Mutational (α^+^)		Total (α^+^)	Deletional		Total (α^0^)	Total frequency (Ethnic group)	Total frequency (Linguistic group)
			-α^3.7^	-α^4.2^	α^CS^	α^PS^		--^SEA^	--^THAI^			
Tai-Kadai	Yong	116 (116; 116)	**0.0388** (0.0179–0.0724)	—	**0.0086** (0.0010–0.0308)	—	**0.0474** (0.0239–0.0833)	**0.0302** (0.0122–0.0612)	—	**0.0302** (0.0122–0.0612)	**0.0776** (0.0466–0.1198)	**0.1125** (0.0910–0.1455) [α^+^ = **0.0845** (0.0630–0.1105), α^0^ = **0.0317** (0.0189–0.0496)]
	Lue	156 (156; 132)	**0.0769** (0.0499–0.1123)	**0.0128** (0.0035–0.0325)	**0.0114** (0.0023–0.0328)	—	**0.1011** (0.0653–0.1410)	**0.0321** (0.0155–0.0582)	—	**0.0321** (0.0155–0.0582)	**0.1332** (0.0909–0.1753)	
	Yuan	48 (48; 18)	**0.0625** (0.0233–0.1311)	—	**0.0556** (0.0068–0.1866)	—	**0.1181** (0.0311–0.2606)	**0.0417** (0.0115–0.1033)	—	**0.0417** (0.0115–0.1033)	**0.1598** (0.0467–0.2950)	
	Shan	53 (53; ND)	**0.1038** (0.0530–0.1781)	—	ND	ND	**0.1038** (0.0530–0.1781)	**0.0377** (0.0104–0.0938)	—	**0.0377** (0.0104–0.0938))	**0.1415** (0.0814–0.2226)	
	Khuen	18 (18; 18)	**0.0556** (0.0068–0.1866)	—	—	—	**0.0556** (0.0068–0.1866)	—	—	—	**0.0556** (0.0068–0.1866)	
Austro-Asiatic	Htin	73 (73; ND)	—	—	ND	ND	-	**0.0137** (0.0017–0.0486)	—	**0.0137** (0.0017–0.0486)	**0.0137** (0.0017–0.0486)	**0.046** (0.0169–0.0963) [α^+^ = **0.0387** (0.0124–0.0862), α^0^ = **0.0077** (0.0002–0.0415)]
	Paluang	19 (19;19)	**0.2105** (0.0955–0.3732)	—	—	—	**0.2105** (0.0955–0.3732)	—	—	—	**0.2105** (0.0955–0.3732)	
	Blang	20 (20; 20)	**0.0250** (0.0006–0.1316)	—	—	—	**0.0250** (0.0006–0.1316)	**0.0250** (0.0006–0.1316)	—	**0.0250** (0.0006–0.1316)	**0.0500** (0.0061–0.1692)	
	Lawa	48 (48;18)	—	—	—	—	—	—	—	—	**0.0000**	
	Mon	34 (34; 9)	**0.0882** (0.0331–0.1822)	—	—	—	**0.0882** (0.0331–0.1822)	—	—	—	**0.0882** (0.0331–0.1822)	
Sino-Tibetan	Skaw Karen	45 (45; ND)	**0.1111** (0.0546–0.1949)	—	ND	ND	**0.1111** (0.0546–0.1949)	—	—	—	**0.1111** (0.0546–0.1949)	**0.1262** (0.0841–0.1794) [α^+^ = **0.1262** (0.0841–0.1794)]
	Pwo Karen	30 (30; ND)	**0.1667** (0.0829–0.2852)	—	ND	ND	**0.1667** (0.0829–0.2852)	—	—	—	**0.1667** (0.0829–0.2852)	
	Padong Karen	28 (28; ND)	**0.1071** (0.0403–0.2188)	—	ND	ND	**0.1071** (0.0403–0.2188)	—	—	—	**0.1071** (0.0403–0.2188)	
	Total	688 (688; 350)	**0.0676** (0.0549–0.0822)	**0.0029** (0.0008–0.0074)	**0.0100** (0.0040–0.0205)	0	**0.0805** (0.0610–0.1026)	**0.0203** (0.0136–0.0293)	0	**0.0203** (0.0136–0.0293)	**0.1008** (0.0788–0.1247)	

ND = Unverified point-mutational alpha-globin gene anomalies (α^CS^, α^PS^) by a dot-blot hybridization method.

*The number of sample enrolled in this study was subjected to four deletional alpha-thalassaemia screening (-α^3.7^, -α^4.2^, --^SEA^ and --^THAI^).

**The number of sample enrolled in this study was subjected to four deletional (-α^3.7^, -α^4.2^, --^SEA^ and --^THAI^) and two mutational (α^CS^, α^PS^) alpha-thalassaemia screening.

**Figure 2:**
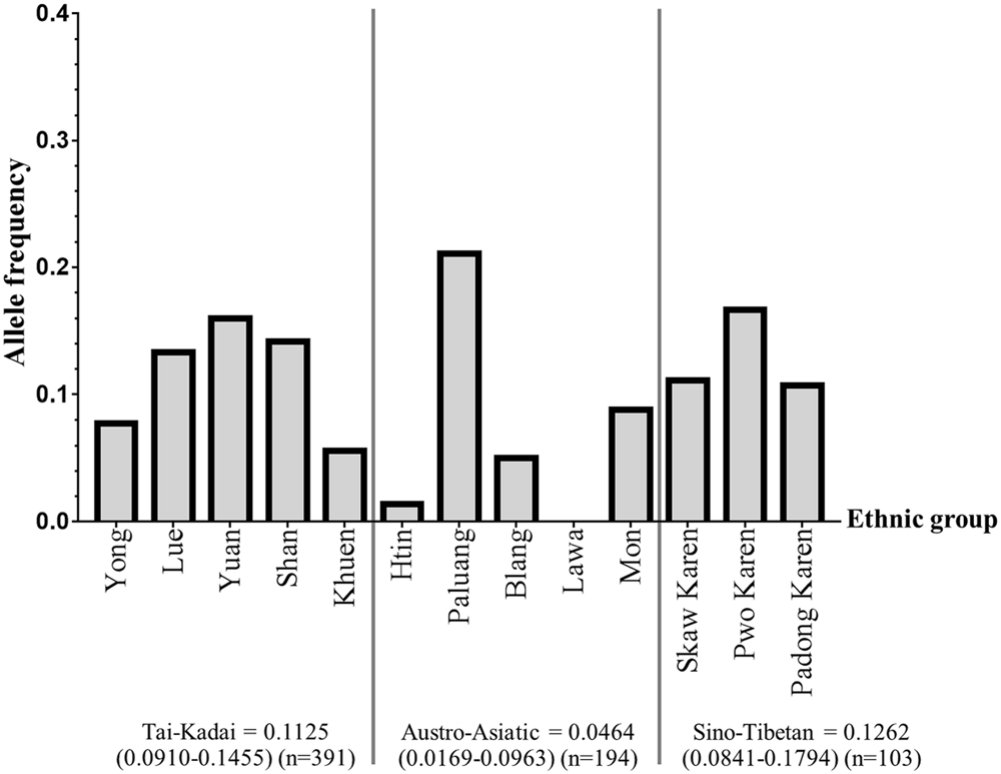
The allele frequency of common α-thalassaemia in each ethnic group. The bar graph represents α-thalassa emia allele frequency. At the bottom of the figure, the total allele frequency of common α-thalassaemia is shown regarding the analysis of the three linguistic groups (Tai-Kadai, Austro-Asiatic and Sino-Tibetan).

The most prevalent deletional α-thalassaemia in the cohort examined was the -α^3.7^ deletion with an allele frequency of 0.0676 (0.0549–0.0822), followed by--^SEA^ and -α^4.2^ at frequencies of 0.0203 (0.0136–0.0293) and 0.0029 (0.0008–0.0074), respectively (Table 2). The presence of non-deletional α-thalassaemia was investigated in 350 samples by a dot-blot hybridization method (Fig. 1b). The non-deletional α^CS^ was detected at an allele frequency of 0.0100 (0.0040–0.0205), and interestingly, this allele was only found in the Tai-Kadai group (Yuan, Lue and Yong) and the mutation was not detected in the Austro-Asiatic groups. The highest α-thalassaemia allele frequency was observed in the Paluang ethnic group [0.2105 (0.0955–0.3732)] while the Lawa showed the lowest α-thalassaemia allele frequency (0.0000) (Table 2 and Fig. 2).

The Sino-Tibetan linguistic group carried the highest frequency of deletional α-thalassaemia [0.1262 (0.0841–0.1794)] followed by Tai-Kadai [0.1125 (0.0910–0.1455)] and the Austro-Asiatic linguistic group [0.0464 (0.0169–0.0963)] (Table 2 and Fig. 2). As noted, analysis of two linguistic groups (Tai-Kadai and Austro-Asiatic) showed that non-deletion α-thalassaemia was found only in the Tai-Kadai linguistic group, giving a frequency of 0.0100 (0.0040–0.0205) (Yong, Lue and Yuan).

The combined analysis of α^+^ and α°-thalassaemia allele frequency may overestimate the incidence of the disease in the population. More importantly, the allele frequency of α°-thalassaemia determines the burden of significant α-thalassaemia syndromes. Therefore, α^+^-thalassaemia was analysed separately from α°-thalassaemia allele frequency. The results showed that α^+^-thalassaemia allele frequency was the highest in the Sino-Tibetan group [0.1262 (0.0841–0.1794)] followed by the Tai-Kadai group [0.0845 (0.0630–0.1105)] and the Austro-Asiatic group [0.0387 (0.0124–0.0862)]. While α°-thalassaemia allele frequency was the highest in the Tai-Kadai group [0.0317 (0.0189–0.0496)] followed by the Austro-Asiatic group [0.0077 (0.0002–0.0415)]. No α°-thalassaemia allele frequency was observed in the Sino-Tibetan group. With regards to ethnicity, the varying allele frequency of α^+^-thalassaemia amongst a variety of ethnic groups ranging from the lowest (0.00) in the Lawa and the Htin, to the highest frequency [0.2105 (0.0955–0.3732)] in the Paluang. While α°-thalassaemia allele frequency was detected in 6 (Yuan, Shan, Lue, Yong, Blang and Htin) out of 13 ethnic groups, the Yuan was the highest [0.0417 (0.0115–0.1033)] (Table 2).

## Discussion

Alpha thalassaemia is a global health problem that is a growing burden^16, 17^, particularly in Southeast Asian ethnic groups. The high prevalence (30%) of α- thalassaemia that has been previously reported in northern Thailand^13, 14, 18^, has been shown to vary from region to region and by ethnic group^15^. However, few studies describing the frequencies of α-thalassaemia in Thai ethnic groups have been conducted, and the data was limited by the small sample size and screening method^9, 14^ (Table 3). Our first survey undertaken using molecular analysis to identify α-thalassaemia amongst 8 ethnic groups had a small sample size^15^, but showed distinct variations between ethnic groups. Furthermore, the population in upper northern Thailand is comprised of a number of ethnic groups which can be categorized into three major linguistic groups. These are comprised of the Tai-Kadai group who are the majority of the present day northern Thai population, the Austro-Asiatic group who are recognized as the descendants of the prehistoric inhabitants of northern Thailand and mostly reside in remote areas, and the hill-tribes group which is comprised of ethnic groups that belong to the Sino-Tibetan and Hmong-Mien linguistic families. From this last group, the Karen ethnic group have the highest population number amongst the hill-tribes of northern Thailand^19, 20^. This is further complicated by the occurrence of diverse genetic backgrounds amongst northern ethnic groups^21, 22^, and therefore the overall incidence of α-thalassaemia from previous surveys might not represent the situation accurately. Thus it was of interest to conduct a larger survey to more accurately determine the real prevalence. Therefore in this study, a larger cohort comprising of individuals from 13 ethnic groups residing in northern Thailand was surveyed for six common α-thalassaemia types. The overall frequency of the six types of α-thalassaemia investigated in this study is 0.1008 (0.0788–0.1247) (Table 2), representing a prevalence of 19.51%) (Table 1). The prevalence data surveyed by this and our previous cohort^15^ are comparable, but lower than previous reports from the general Thai population that reported the prevalence of α-thalassaemia at 26.42% (28/106)^9^ (Table 3).

**Table 3:** Previous reports of α-thalassaemia prevalence in the population residing in northern Thailand.

Author, year	No. samples	No. affected	Alpha-thalassaemia allele frequency							Total frequency
			-α^3.7^	-α^4.2^	--^SEA^	--^THAI^	α^CS^	α^PS^	ααα	
Hundrieser *et al*., 1988	106	28	0.0943	0.0047	0.0236	—	—	—	0.0142	0.1368
Lemmens-Zygulska *et al*., 1996	215	77	0.0977	—	0.0698	—	0.0116	—	0.0070	0.1860
Lithanatudom *et al*., 2016	141	33	0.0922	—	0.0177	—	0.0106	—	—	0.1206
This study	688	124	0.0676	0.0029	0.0203	—	0.0100	—	—	0.1008

In accordance with our previous study^15^ and the findings of other studies^14, 16, 18^, the -α^3.7^ deletion is the most common α-thalassaemia present amongst Thais and Thai ethnic groups, followed by --^SEA^ ^6, 7^. Interestingly, the heterozygous --^SEA^ deletion is very common in the Tai-Kadai linguistic group. The data is also consistent with a study undertaken in the Yunnan province of Southwestern China which showed that the --^SEA^ deletion type is the most common α-thalassaemia^23^ and supports evidence that the Tai-Kadai speaking people staying in Northern Thailand migrated there from the southwest of China^24, 25^. It also supports the genetic diversity of this abnormal gene between population groups.

Similarly, evidence for the genetic diversity of this gene was found with the α^CS^ mutation. While the overall allele frequency of the α^CS^ allele was 0.0100 (0.0040–0.0205) (Table 2), it was only found in the Tai-Kadai linguistic group (Yuan, Lue and Yong) and the mutation was not detected in the Austro-Asiatic groups. However, α^CS^ is the most prevalent α-globin variant in the Southeast Asian population^26^ and while people with heterozygous α^CS^ have an almost normal clinical presentation, when inherited in a compound heterozygous state along with α^0^-thalassaemia, a more severe presentation than deletional Hb H disease can occur^26^. In contrast to the native Thai population, we did not find the --^THAI^ deletion or the Hb Pakse α-globin variant in this cohort. This latter observation is in accordance with an earlier study which showed that Hb Pakse was not found in the population residing in northern Thailand^3^.

With regards to the frequency of α-thalassaemia observed in each linguistic group, this study detected considerable variation amongst the different ethnic groups. The highest frequency of α-thalassaemia [0.1262 (0.0841–0.1794): α^+^ = 0.1262 (0.0841–0.1794), α^0^ = 0] was seen in the Sino-Tibetan (Karen) linguistic group and the -α^3.7^ deletion type was the only α-thalassaemia type existing in this group. Importantly, the Paluang, Karen and Shan ethnic groups showed a very high frequency of the -α^3.7^ deletion type. Since these three ethnic peoples live along the Thailand-Myanmar border^27^ which is a malaria endemic area^28^, the high frequency of the -α^3.7^ may reflect natural selection due to protection against severe malaria infection^29^. Moreover, the presence of the -α^3.7^ deletion in all three Karen ethnic groups (Skaw, Pwo and Padong) is at very similar levels, supporting the common origin of these ethnic groups, and showing that the Karen seem to have a homogenous genetic background. The frequency of α-thalassaemia is also high in the Tai-Kadai group [0.1125 (0.0910–0.1455): α^+^ = 0.0845 (0.0630–0.1105), α^0^ = 0.0317 (0.0189–0.0496)]. Interestingly, this linguistic group shows the highest frequency of heterozygous α-thalassaemia 1 (--^SEA^) which is characterized by deletion of two α-globin genes, and this was supported by the detection of one individual with Hb H disease in this group. In contrast to the other ethnic groups in the Tai-Kadai linguistic group, the Lue show significant gene diversity with 4 types of α-thalassaemia detected in this ethnic group. This is likely to be the result of a founder effect and/or inter-ethnic marriage between the Lue and other ethnic groups during their migration through Laos. The predominance of --^SEA^ and α^CS^ types in Tai-Kadai linguistic group elevates their risk of conceiving fetuses with Hb Bart’s hydrop fetalis or Hb H-CS disease. The lowest frequency was recorded in Austro-Asiatic linguistic group since no α-thalassaemia was detected in any of the 48 Lawa people investigated.

## Conclusion

Our study presents the results of the screening of a large cohort representing 13 ethnic groups from northern Thailand for α-thalassaemia. As the prevalence of α-thalassaemia is relatively high and the majority of these groups are still unaware of their thalassaemia status, couples who are members of particular ethnic populations at risk for α°-thalassaemia (--^SEA^, --^THAI^) such as the Yuan, Shan, Lue and Yong should be recommended for haematological screening prior to planning for pregnancy to control the severe types of α-thalassaemia. Future studies might be directed to study the whole α-globin locus in order to determine whether novel α-globin gene abnormalities may exist that are unique to a particular ethnic group.

## Materials and Methods

### Study populations

**Figure 3:**
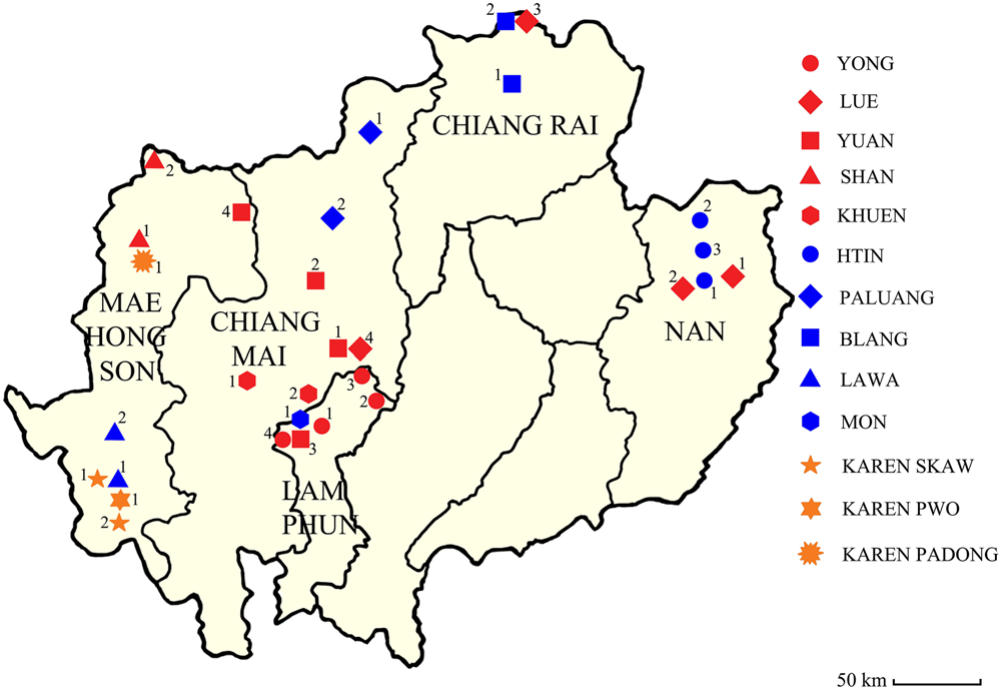
Geographical map representation of sampling areas and distribution of the ethnic groups residing in northern Thailand. The red colour represents Tai-Kadai speaking ethnic groups, the blue colour represents the Austro-Asiatic speaking ethnic groups and orange indicates the Sino-Tibetan speaking ethnic groups. This figure was modified using the Photoshop program. The original source of this figure can be found at https://commons.wikimedia.org/wiki/File:Thailand_location_map.svg which is licensed under the “Creative Commons Attribution 3.0 Unported” that is free to share (to copy, distribute and transmit the work) and remix (to adapt the work).

Northern Thailand has 18 officially recognized ethnic populations^19, 20^. For this survey samples were obtained from 13 ethnic groups from 30 villages distributed in five provinces of northern Thailand. The cohort comprised (a) 278 newly genotyped samples and (b) 269 subjects previously genotyped for hemoglobin E for whom α-thalassaemia genotype has not been reported^30^. In addition (c) α-thalassaemia genotypic data from 141 subjects as previously reported^15^ was included, giving a total 688 samples. The criteria for population sampling was as described elsewhere^22, 24, 30, 31^. Briefly, all volunteers enrolled in this study were healthy, over 20 years of age, unrelated, and recognized as a member of the study ethnic population for at least three generations with no admixture from other populations. The designed number of sample size enrolled in this study was 30 samples per ethnic group. Although some difficulties arose in obtaining appropriate number of samples from some ethnic groups such as the Padong Karen who practice endogamous marriage, the Palaung and Blang who have small population sizes and the Khuen who traditionally marry with people from other ethnic groups (interethnic marriage) giving offspring (admixed population) that cannot be recruited for this study, the sample sizes from such mentioned ethnic groups are still nearly in the power of calculation for population analyses as stated by Jobling *et al*., 2013 (20–50 individuals per populations are recommended)^32^. The location of sampling areas and details are shown in Table 4 and Fig. 3. All subjects from categories (a) to (c) were enrolled after informed consent. Ethical approval of all methods and experimental protocols according to the guidelines was follows: the Yong ethnic group (a) and all subjects of category (b) were approved by the Human Experimentation Committee, Research Institute for Health Sciences, Chiang Mai University, Thailand. All subjects of category (c) were approved by the Policy Review Board of the Pan Asia SNP consortium as described elsewhere^33^. It should be noted that both the Lue and the Htin ethnic population samples of category (a) were collected more than 10 years ago and therefore oral informed consent was implemented with the assistance of the head of each village.

**Table 4:** Linguistic group, ethnicity, location and number of samples of the 13 ethnic groups.

Linguistic group	Ethnic group	Location (district, province)	Locality (latitude °N/ Longitude °E)	Number of sample
Tai-Kadai	Yong	Pa Sang, Lamphun Pa	18°53′/98°91′	65
		Sang, Lamphun*	18°44′/98°90′	20
		Mae Tha, Lamphun	18°50′/99°17′	2
		Ban Thi, Lamphun	18°69′/99°15′	1
		Ban Hong, Lamphun	18°33′/98°81′	28
	Lue	Pua, Nan	19°14′/100°93′	40
		Pua, Nan*	19°17′/100°91′	1
		Tha Wang Pa, Nan	19°08′/100°77′	35
		Mae Sai, Chiang Rai	20°41′/99°95′	38
		Doi Sa Ket, Chiang Mai	18°89′/99°12′	24
		Doi Sa Ket, Chiang Mai*	18°89′/99°12′	18
	Yuan	San Sai, Chiang Mai*	18°85′/99°04′	7
		Mae Taeng, Chiang Mai*	19°12′/98°93′	9
		Ban Hong, Lamphun*	18°30′/98°81′	2
		Pai, Mae Hong Son	19°44′/98°50′	30
	Shan	Muang, Mae Hong Son	19°29′/97°96′	23
		Pang Ma Pha, Mae Hong Son	19°62′/98°11′	30
	Khuen	Mae Wang, Chiang Mai*	18°62′/98°77′	12
		San Pa Tong, Chiang Mai*	18°62′/98°89′	6
Austro-Asiatic	Htin	Pua, Nan	19°08′/100°55′	25
		Thung Chang, Nan	19°23′/100°52′	23
		Chiang Klang, Nan	19°19′/100°54′	25
	Paluang	Fang, Chiang Mai*	19°92′/99°21′	11
		Chiang Dao, Chiang Mai*	19°36′/98°96′	8
	Blang	Mae Chan, Chiang Rai*	20°14′/99°85′	12
		Mae Sai, Chiang Rai*	20°43′/99°87′	8
	Lawa	Mae La Noi, Mae Hong Son*	18°23′/97°56′	18
		Mae Sa Rieng, Mae Hong Son	18°16′/97°94′	30
	Mon	Pa Sang, Lamphun	18°52′/98°89′	25
		Pa Sang, Lamphun*	18°52′/98°89′	9
Sino-Tibetan	Skaw Karen	Mae Sa Rieng, Mae Hong Son	18°20′/97°88′	31
		Sob Mei, Mae Hong Son	18°01′/97°88′	14
	Pwo Karen	Mae Sa Rieng, Mae Hong Son	18°15′/97°93′	30
	Padong Karen	Muang, Mae Hong Son	19°14′/97°93′	28
		Total		688

*Previously reported groups screened for 6 types of deletion and point mutation of α-thalassaemia gene for comparison^15^.

### DNA extraction

Five milliliters of peripheral blood from human subjects was collected after individual informed consent, and total genomic DNA was extracted using an inorganic salting out protocol as described elsewhere^34^. Quality and quantity of extracted genomic DNA from all samples were examined by 1% agarose gel electrophoresis and spectrophotometry (OD_260_/OD_280_). All samples were kept at -20 °C until use.

### Multiplex gap-PCR analysis of the deletional alpha globin gene

The four most common deletional α-thalassaemias (-α^3.7^, -α^4.2^, --^SEA^ and --^THAI^) in the Thai population were investigated in this study. All samples were genotyped for the four deletional types of α-thalassaemia by multiplex gap-polymerase chain reaction modified from Chong and colleagues^35^. Briefly, nine specific primers were used in PCR reaction, consisted of primers α2/3.7-F, 3.7-R, α2-R, 4.2-F, 4.2-R, SEA-F, SEA-R, THAI-F and THAI-R. Each PCR reaction was performed in a single tube for simultaneous amplification of different amplicons under an initial denaturation at 95 °C for 15 minutes and followed by thirty-five cycles of denaturation for 45 second at 98 °C, annealing at 60 °C for 1.30 minute, extension at 72 °C for 2.15 min with an additional final extension at 72^o^C for 5 min after the last cycle. PCR products were analysed by 1.5% agarose gel electrophoresis compared with positive controls as shown in Fig. 1a. To ensure the genotyping accuracy of the multiplex gap-PCR, every single round of PCR amplification of unknown samples was performed paralleled with the positive controls (Fig. 1a, lane 1, 2, 3 and 4 are --^THAI^/αα, --^SEA^/αα, -α^4.2^/αα and -α^3.7^/αα, respectively) and a negative control (lane 5 is genotyped as αα/αα). DNA samples from each unknown individual was genotyped at least in duplicate.

### Dot-blot hybridization analysis of the mutational alpha globin gene

A total of 350 samples were screened for two common types of non-deletional α-thalassaemia (α^CS^ and α^PS^). The dot-blot hybridization method was employed as described elsewhere^36^. The α-globin gene was amplified by PCR using primers αF and α2 R. The 331 bp-PCR products were validated by 1.5% agarose gel electrophoresis and were then subsequently hybridized with specific probes for α^CS^ and α^PS^ as well as a normal probe. The resulting genotype of each unknown sample was interpreted in parallel with controls, which consisted of a normal sample and homozygous α^CS^ and α^PS^. A blue spot was interpreted as a positive signal (Fig. 1b). The genotyping quality of the dot blot hybridization of unknown samples was ensured by controls (Fig. 1b, positive controls are samples with known genotype of α^CS^ homozygous and α^PS^ homozygous while the negative control was αα/αα). Unknown samples were always tested in paralleled with controls, and analysis was conducted in duplicate.

### Statistical Methods

All the allele frequencies were calculated using the Microsoft Excel program (version 2016, Microsoft Corporation, USA) with the function BinomLow and BinomHigh (add-ins) derived from JavaStat to compute the exact binomial confidence interval (95%). The bar graph was generated by the PRISM software (version 7.00, GraphPad Software, Inc. USA).

### Data availability statement

The data sets generated and analysed during the current study are available within the paper.

## Electronic supplementary material

Supplementary Information
