# Supplementary material for: A comprehensive ethnic-based analysis of alpha thalassaemia allelle frequency in northern Thailand
Source: Sci Rep. 2017 Jul 5;7:4690. doi: 10.1038/s41598-017-04957-2 (PMC5498591; doi:10.1038/s41598-017-04957-2)
Supplement: Supplementary file 1 — Supplementary Information [file 41598_2017_4957_MOESM1_ESM.pdf]

Supplementary Information

A comprehensive ethnic-based analysis of alpha thalassaemia allelle frequency in northern Thailand

Mattapong Kulaphisit,[1] Jatupol Kampuansai,[1] Kamonlak Leecharoenkiat,[2] Methi Wathikthinnakon,[1] Daoroong Kangwanpong,[1] Thongperm Munkongdee,[4] Saovaros Svasti,[4] Suthat Fucharoen,[4] Duncan R. Smith [3] and Pathrapol Lithanatudom [1*]

[1] Department of Biology, Faculty of Science, Chiang Mai University, Chiang Mai, 50300, Thailand.

[2] Department of Clinical Microscopy, Faculty of Allied Health Sciences, Chulalongkorn University, Bangkok, 10330, Thailand.

[3] Molecular Pathology Laboratory, Institute of Molecular Biosciences, Mahidol University, Nakornpathom, 73170, Thailand

[4] Thalassemia Research Center, Institute of Molecular Biosciences, Mahidol University, Nakornpathom, 73170, Thailand.

[*]Correspondence and requests for materials should be addressed to P.L. (email: pathrapol_li@hotmail.com)

Supplementary figures

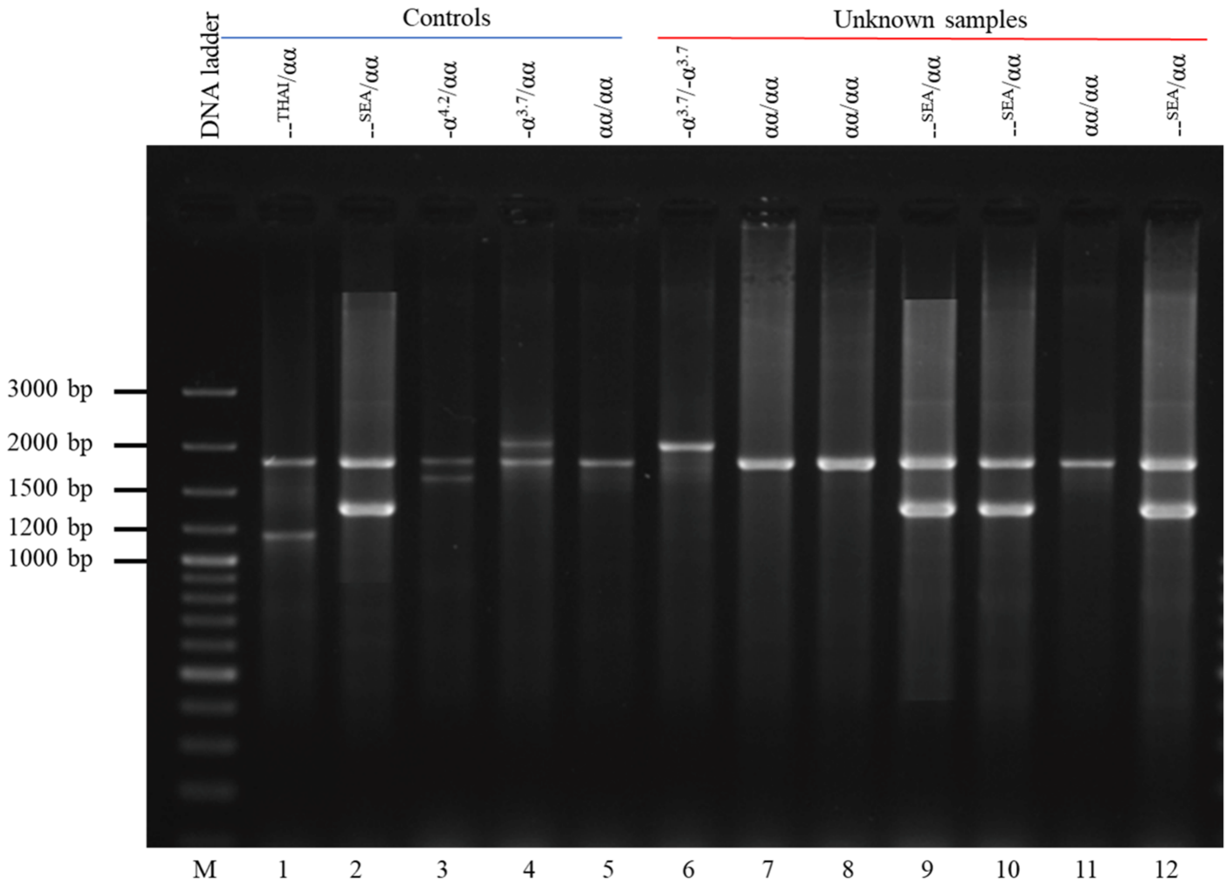

Supplementary Figure S1. PCR product of deletional α-thalassaemia analysed using the multiplex-gap PCR. M = DNA marker, lane 1-4 = positive control of alpha-globin heterozygotes which are $--^{THAI}/\alpha\alpha$, $--^{SEA}/\alpha\alpha$, $-\alpha^{4.2}/\alpha\alpha$ and $-\alpha^{3.7}/\alpha\alpha$ respectively, lane 5 = negative control, lane 6 = unknown sample genotyped as $-\alpha^{3.7}$ homozygote, lane 7-8 and 11 = unknown samples genotyped as normal, lane 9-10 and 12 = unknown samples genotyped as $--^{SEA}$ heterozygotes. No overexposure and high-contrast are applied to the gel. The cropped gel is employed in the main figure (Figure 1a).

Sample list

TL-146	TL-150	TL-151	TL-157	TL-159	TL-160	TL-162	TL-164	TL-167	TL-169	Positive control Normal
TL-180	TL-201	TL-202	TL-208	TL-210	TL-211	TL-212	TL-216	TL-218	TL-220	Positive control HbCS
TL-221	TL-222	TL-223	TL-228	TL-230	TL-231	TL-234	TL-235	TL-238	TL-239	Positive control HbPS

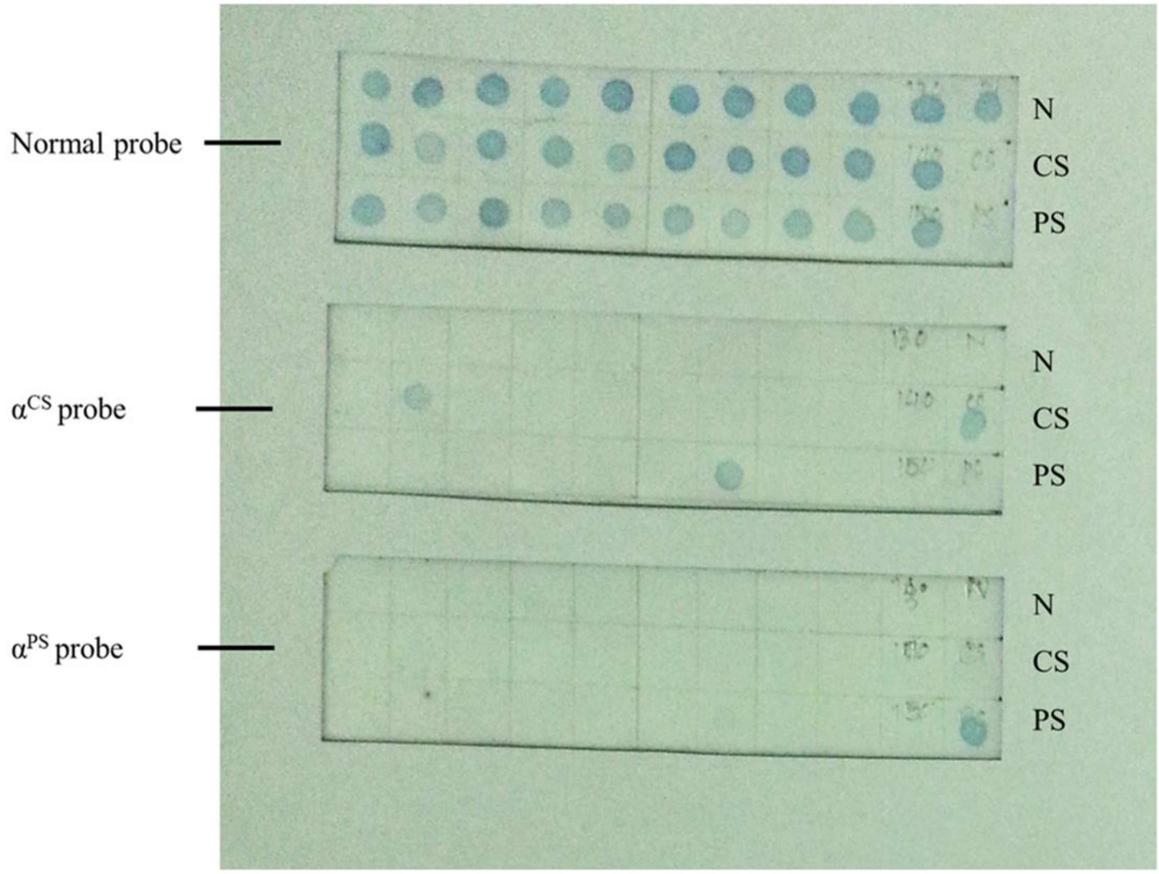

Supplementary Figure S2. Dot-blot hybridization analysis of samples from The Lue ethnic group. Samples TL-201 and TL-234 were genotyped as α^{CS} heterozygotes. No samples were positive for α^{PS}. No overexposure and high-contrast are applied to the blot. The cropped blot is employed in the main figure (Figure 1b).
